# Are Exercise Interventions for People With Knee Osteoarthritis Dosed Appropriately to Meet the World Health Organisation's Physical Activity Guidelines?

**DOI:** 10.1002/msc.70089

**Published:** 2025-03-23

**Authors:** Titus E. Zhao, Matthew D. Jones, Mitchell T. Gibbs

**Affiliations:** ^1^ School of Clinical Medicine Faculty of Medicine and Health University of New South Wales Sydney Australia; ^2^ School of Health Sciences Faculty of Medicine and Health University of New South Wales Sydney Australia; ^3^ Centre for Pain IMPACT Neuroscience Research Australia Sydney Australia

## Abstract

**Objective:**

This study aimed to determine the number and proportion of exercise interventions within preexisting clinical trials for people with knee osteoarthritis (KOA) that satisfied the World Health Organisation's (WHO) guidelines for physical activity.

**Methods:**

A descriptive analysis of studies included in an umbrella review was undertaken. Data from each exercise intervention relating to the type, dose and intensity of exercise was extracted, and the number and proportion of interventions that satisfied the WHO guidelines (aerobic, muscle strengthening, balance [for studies where the average age was more than 65 years old], a combination or all) was recorded at the study and intervention level.

**Results:**

Data were extracted from 199 studies containing 266 exercise interventions. Overall, only one study (0.5%) satisfied all components of the WHO guidelines. Of the 122 interventions that had an average participant age over 65, none fulfiled all aspects of the WHO guidelines, which included balance. There were 16 (6.0%) and 12 (4.5%) other interventions that satisfied the aerobic or muscle strengthening components of the guidelines, respectively.

**Conclusion:**

This descriptive analysis highlighted the lack of exercise interventions in clinical trials for people with KOA that satisfied the WHO guidelines. Thus, they may not be dosed appropriately to achieve broader health outcomes associated with following the physical activity guidelines.

## 1 Introduction

Exercise is a core treatment for people with knee osteoarthritis (KOA) (Arden et al. 2021) and has been shown to reduce pain, increase physical function, and improve overall quality of life (B. Lawford et al. 2024). However, the effects of exercise on outcomes such as pain and disability are often small and comparable to other non‐active, guideline concordant treatment modalities such as massage, manual therapy, and analgesic medications (Abbott et al. 2013; Weng et al. 2023). Interestingly, exercise as a modality in chronic pain conditions is often claimed as a more beneficial choice due to the ability to result in positive health‐related outcomes compared to passive interventions (Gibbs et al. 2024). Indeed, management/prevention of non‐communicable disease is a priority for practitioners to consider in the overall management of KOA with higher documented rates of cardiovascular disease including an estimated 20% higher risk of myocardial infarction and 29% for stroke (Park et al. 2023). Moreover, the presence of these co‐morbidities alongside KOA is associated with worse outcomes (Larsen and Elsoe 2025; Muckelt et al. 2020). It has also recently been shown that higher compliance to the American College of Sports Medicine's exercise prescription guidelines does not influence clinical outcomes (pain and disability) in KOA (B. J. Lawford et al. 2024), which suggests basing exercise dose on the potential to influence these outcomes may make little difference. Conversely, the effects of exercise on managing multimorbidity, including KOA, are well established with demonstrated effects on health‐related quality of life, physical function, anxiety and depression (Bricca et al. 2020). Thus, exercise appears a pragmatic option for people with KOA, displaying similar effects to other interventions on clinical outcomes but with the additional ability to manage/reduce the risk of multimorbidity, which is apparent in this population.

The World Health Organisation's (WHO) Guidelines on Physical Activity and Sedentary Behaviour (Bull et al. 2020) are global recommendations aimed at improving overall health and mitigating health‐related risks. The benefits of meeting these guidelines on improving health‐related outcomes were consistently demonstrated throughout the literature (Martinez‐Gomez et al. 2024; Nyberg et al. 2020; Sun et al. 2014). However, according to a systematic review and meta‐analysis of over three million participants, only one in five adults meet the physical activity guidelines for aerobic and muscle strengthening activity (Garcia‐Hermoso et al. 2023). Furthermore, it has been reported that only a small to moderate proportion of people with KOA meet these guidelines (Wallis et al. 2013), which may be influenced by a range of factors including symptoms and demographic factors (Stubbs et al. 2015). Indeed, it is plausible that the reduced rates of adherence to physical activity in people with KOA may partly explain the higher rates of observed co‐morbidities (Park et al. 2023). Thus, it is important to understand how exercise for KOA is being delivered to identify if these individuals are receiving interventions that are appropriate for the management/prevention of such co‐morbidities.

A recent audit of the Cochrane review of exercise for chronic low back pain showed that very few exercise interventions are appropriately dosed to achieve broader health benefits based on the WHO physical activity guidelines (Gibbs et al. 2024). Within the chronic low back pain literature, there does not appear to be a superior mode or dose of exercise that leads to improved outcomes such as pain and disability (Hayden et al. 2021). Additionally, people living with chronic low back pain also display higher rates of cardiovascular disease compared with asymptomatic individuals (Williams et al. 2018). These characteristics, no clear superior mode of exercise for improving clinical outcomes and an increased rate of multimorbidity, share similarities with KOA (with the caveat of favouring land‐based exercise) (B. Lawford et al. 2024). These findings suggest a critical need to re‐examine the basis for exercise prescription in conditions where there is: a) no superior mode of exercise leading to clinically meaningful improvements in outcomes and b) an identified health disparity between those living with the condition and asymptomatic counterparts and/or people living with the condition plus additional co‐morbidities. Therefore, this study aimed to explore whether current exercise interventions for KOA are appropriately dosed (based on the WHO physical activity guidelines) for health‐related benefits. The aim was to determine the proportion of interventions in randomised controlled trials (RCTs) of exercise for people with KOA that satisfy the minimum components of the WHO physical activity guidelines.

## 2 Methods

### 2.1 Data Sources, Searches and Study Selection

This study was a descriptive analysis of RCTs included in an umbrella review of exercise for hip and KOA (Varongot‐Reille et al. 2024). The umbrella review was a comprehensive review of exercise for KOA and included 41 meta‐analyses comprising 238 unique RCTs. All RCTs in the umbrella review were included in our descriptive analysis provided they included people with KOA. The protocol for this paper was pre‐registered on the Open Science Framework (https://osf.io/jtwq3/).

### 2.2 Data Extraction

Data were extracted from each RCT into a purpose‐built electronic spreadsheet (Microsoft Excel). The spreadsheet was initially piloted and reviewed by members of the research team, where variables for data extraction were added and modified. Variables from each study that were extracted included study characteristics (e.g., author and year), participant characteristics (e.g., age), and details of the exercise intervention based on the FITT principle (frequency, intensity, time, and type), as per previous research (Gibbs et al. 2024).

Listed below are the WHO physical activity guidelines for adults aged 18–64 years (Bull et al. 2020):

1. All adults should undertake regular physical activity.
2. Adults should do at least 150–300 min of moderate‐ and vigorous‐intensity aerobic physical activity, or at least 75–150 min of vigorous‐ and vigorous‐intensity aerobic physical activity or an equivalent combination of moderate‐ and vigorous‐intensity activity throughout the week for substantial health benefits.
3. Adults should also perform muscle‐strengthening activities at moderate or greater intensity that involve all major muscle groups on 2 or more days a week, as these provide additional health benefits.
4. Adults may increase moderate‐ and vigorous‐intensity aerobic physical activity to more than 300 min, or do more than 150 min of vigorous‐intensity aerobic physical activity or an equivalent combination of moderate‐ and vigorous‐intensity activity throughout the week for additional health benefits.

For adults aged 65 years and older, there is an additional guideline (Bull et al. 2020):

5. As part of their weekly physical activity, older adults should perform varied multicomponent physical activity that emphasises functional balance and strength training at moderate or greater intensity, on 3 or more days a week, to enhance functional capacity and prevent falls.

The WHO guidelines report exercise intensity as ‘moderate’ or ‘high’; however, intensity can be classified in various ways. Thus, studies that reported the intensity of their exercise intervention(s) based on heart rate or the Bourg rating of perceived exertion scale were stratified into light, moderate, or high intensity based on established thresholds (Norton et al. 2010). Interventions involving weight/resistance exercises that reported intensity through repetition maximums (RM) were compared to the American College of Sports Medicine Position Stand (Garber et al. 2011). They were then similarly classified as light, moderate, or high intensity. It was assumed that ‘maximal voluntary contraction’ and ‘peak torque’ were equivalent to RM. If studies reported the intensity of the intervention in multiple ways, the objective measure (e.g., heart rate maximum, heart rate reserve, RM) was preferentially used over the subjective measure (e.g., rating of perceived exertion). For studies where the average age of the included participants was over 65 years, data were also extracted relating to any balance component of the exercise intervention, consistent with the additional guidelines relating to this age group. All data were extracted by two reviewers. Issues or disagreements were resolved by discussion with a third reviewer when necessary.

### 2.3 Data Synthesis and Analysis

Data were extracted from each study according to the FITT principle and mapped against the WHO physical activity guidelines as outlined in Table 1.

**TABLE 1:** WHO guidelines in terms of type, dose, and intensity.

WHO guidelines on physical activity			
Type	Aerobic exercise	MSE	Balance exercise (+65 years only)
Dose	≥ 75–150 min a week (depending on intensity)	≥ 2 times a week for the whole body	≥ 3 times a week
Intensity	Moderate ( ≥ 150 min) or vigorous ( ≥ 75 min)	≥ Moderate	≥ Moderate

Abbreviation: MSE = muscle strengthening exercise.

The counts and proportions of exercise interventions containing aerobic, muscle strengthening, balance (+65 years only) or a combination of these were recorded. Throughout extraction, it was noted if an intervention met the following:

– Type only (e.g., an aerobic intervention but the dose was < 150 min moderate activity/75 min vigorous activity, or a combination of the two)
– Type and dose (e.g., an aerobic intervention meeting the required minutes at a lower than moderate intensity OR where intensity was not adequately reported)
– Type, dose, and intensity (e.g., an aerobic intervention meeting all components of the guideline)
– All guidelines (i.e., an intervention meeting both the aerobic and muscle strengthening guidelines OR an intervention meeting the aerobic, muscle strengthening, and balance guidelines in an intervention where the average age of participants was > 65 years).

Each intervention in each included study was compared to the criteria listed in Table 1, meaning if a single study reported multiple exercise interventions/groups, all were assessed against the WHO physical activity guidelines. Therefore, data was analysed and reported at both the study and group level to allow for studies including multiple groups. Further, interventions were not double counted within the analysis. As such, if an intervention met both (or all three guidelines in the case of average age > 65 years), it was not recorded as satisfying individual guidelines separately.

### 2.4 Protocol Deviations

Fulfilment of criteria could not be accurately determined due to numerous studies (*n* = 41) reporting a range of doses and intensities throughout the timeframe of their intervention. This was because in most of these studies, exercise dosage progressed over the programme to allow participants to gradually adjust to the exercise load, especially if they were previously inactive. To remedy this issue, the median of the frequency, volume, and intensity (where quantitatively reported) was taken.

## 3 Results

This review included 199 RCTs containing 266 exercise interventions. General details of the studies can be found in the original umbrella review (Varongot‐Reille et al. 2024). Thirty‐seven studies from the umbrella review were excluded from this study due to cases where the full text of the study or the intervention was not able to be found (*n* = 11) or translated (*n* = 8), interventions were duplicated in another included study of the same trial (*n* = 16), the study was a thesis dissertation (*n* = 1) or the study was retracted (*n* = 1). The adjusted average age for participants in the included RCTs in this study was 64.4 ± 5.8.

Table 2 shows the results of all included studies that met the aerobic and/or muscle strengthening WHO physical activity guidelines. Table 3 shows the subset of studies (*n* = 104) and interventions (*n* = 122) with participants over the age of 65 that included the additional guideline of balance and if they satisfied the guidelines for each component and/or combinations of components. As shown in Table 2, only one intervention holistically met the guidelines. Table 3 shows that for studies with an average participant age over 65 years and with further consideration of the balance component, no study/intervention met the entirety of the WHO physical activity guidelines. Only 16 (6.0%) and 12 (4.5%) of interventions fulfiled the aerobic or muscle strengthening component of the guidelines, respectively, and only one (0.8%) of the applicable 122 interventions met the balance component guidelines. 60 aerobic interventions (50.8%) and 165 (75.7%) muscle strengthening interventions did not adequately report intensity, which limits the ability to examine these interventions against the guidelines. Based on our protocol and previous work (Gibbs et al. 2024), these interventions were deemed to not satisfy the intensity component of the guidelines.

**TABLE 2:** Exercise interventions meeting the WHO physical activity guidelines at the group and study level.

	None	Aerobic only	MSE only	Both
Group level (*n* = 266)	237 (89.1%)	16 (6.0%)	12 (4.5%)	2 (0.8%)
Study level (*n* = 199)	183 (92.0%)	9 (4.5%)	6 (2.3%)	1 (0.5%)

Abbreviation: MSE = muscle strengthening exercise.

**TABLE 3:** Exercise interventions with an average age > 65 meeting the WHO physical activity guidelines at the group and study level.

	None	Aerobic only	MSE only	Balance only	Balance + aerobic	Balance + MSE	Aerobic + MSE	All
Group level (*n* = 122)	103 (84.4%)	10 (8.2%)	9 (7.4%)	1 (0.8%)	0 (0%)	0 (0%)	0 (0%)	0 (0%)
Study level (*n* = 104)	96 (92.3%)	3 (2.9%)	4 (3.8%)	1 (0.5%)	0 (0%)	0 (0%)	0 (0%)	0 (0%)

Abbreviation: MSE = muscle strengthening exercise.

Counts of interventions at the study and group levels that met the exercise type, regardless of dose, are presented in Table 4. Interventions at the study and group level meeting exercise type and dose irrespective of intensity are shown in Table 5. A total of 60 studies (30.2%) and 82 interventions (30.8%) contained the correct type of exercise; however, only two studies (1.0%) and four interventions (1.5%) met the dose requirements of the exercise types in the WHO guidelines.

**TABLE 4:** Exercise interventions that contained an exercise type consistent with the WHO physical activity guidelines at the group and study level.

	None	Aerobic only	MSE only	Both
Group level (*n* = 266)	12 (4.5%)	36 (13.5%)	136 (51.1%)	82 (30.8%)
Study level (*n* = 199)	19 (9.6%)	23 (11.6%)	97 (48.7%)	60 (30.2%)

Abbreviation: MSE = muscle strengthening exercise.

**TABLE 5:** Exercise interventions that contained both an exercise type & dose consistent with the WHO physical activity guidelines at both the group and study level.

	None	Aerobic only	MSE only	Both
Group level (*n* = 266)	188 (70.7%)	52 (19.6%)	22 (8.3%)	4 (1.5%)
Study level (*n* = 199)	151 (75.9%)	34 (17.1%)	12 (6.0%)	2 (1.0%)

Abbreviation: MSE = muscle strengthening exercise.

## 4 Discussion

This descriptive analysis revealed that very few exercise interventions tested in clinical trials for KOA meet the WHO physical activity guidelines. Only one study (0.5%) contained two‐groups (0.8%) that met all aspects of the guidelines where the mean age of participants was below 65 years (i.e., not including a balance component). Conversely, no studies with an average participant age over 65 years met the guidelines, which included the balance component. Interestingly, only one intervention from this sub‐set of studies met the balance requirements of the guidelines. When the guidelines were explored individually, 16 (6.0%) interventions met the aerobic guidelines and 12 (4.5%) met the muscle strengthening guidelines, respectively. Without the consideration of intensity (which was often not reported), the majority of interventions (188, 70.7%) did not meet either the aerobic or muscle strengthening type and dose guidelines. Of note, the majority of studies (90.4%) did report a type of exercise consistent with the guidelines, though failed to satisfy either the dose or intensity component and this was often poorly reported. Indeed, it is plausible that the counts of studies/interventions meeting the guidelines may be confounded by poor reporting, especially regarding intensity, which is a common problem in this field of research (Hansford et al. 2022).

The findings of this study are consistent with the broader literature examining exercise interventions against the WHO physical activity guidelines in musculoskeletal conditions. For example, a recent study investigating exercise interventions for chronic low back pain (Gibbs et al. 2024) presented similar results, finding a minimal number of exercise interventions meeting the WHO physical activity guidelines. However, unlike the review of chronic low back pain, this study also further considered the addition of balance within the physical activity guidelines for individuals aged 65 and over. This was particularly relevant with the mean age of participants in the RCTs included in this study being significantly higher at 64.4 compared to the compared to the chronic low back pain study, which reported an average age of 43.7. Adding the balance criteria allowed the present study to consider all components of the WHO physical activity guidelines, which highlighted the lack of interventions in this age group considering the balance component. Indeed, while the proportions of interventions meeting the WHO physical activity guidelines reported in this study is overwhelmingly low and comparable to the previous review, it is important to note this is often not the target of these interventions within the literature.

The findings of this study suggest that the largest gap in meeting the WHO physical activity guidelines in KOA is most evident in muscle strengthening exercise. Whilst 218 exercise interventions (82.0%) contained a muscle strengthening component, only 26 of them (9.8%) met the dose requirement (i.e., muscle strengthening exercise involving all major muscle groups on 2 or more days a week). The most common deviation from this guideline was interventions failing to prescribe whole‐body exercise but rather localising it to the lower limb. Interestingly, despite lower limb strengthening exercise being widely prescribed in trials for KOA (B. Lawford et al. 2024)improvements in strength of the lower limb in people with KOA appear to only explain a small portion of improvement in clinical outcomes (pain and disability; Runhaar et al. 2023). Conversely, engaging in whole‐body resistance training appears to have numerous systemic health‐related benefits, including reduced blood pressure, resting low‐density lipoprotein, and HbA 1C (Westcott 2012), which are all risk factors for cardiovascular disease (Canto and Iskandrian 2003). Further, based on evidence showing that a concordant exercise dose does not impact clinical outcomes (B. J. Lawford et al. 2024) it is unlikely that a whole‐body muscle strengthening programme will produce inferior outcomes compared to lower‐limb only. Taken together, it appears engaging in whole‐body strengthening may be a superior alternative for people with KOA due to the potential secondary benefits, whilst not losing the benefit of lower limb strengthening exercise on improvements in pain and disability, albeit a small effect (B. Lawford et al. 2024).

As mentioned, people with KOA tend to be physically inactive (Wallis et al. 2013). One viable strategy to appropriately dose exercise for sedentary individuals living with KOA may be to progressively increase the frequency, volume, and intensity of exercise throughout an intervention towards the WHO physical activity guidelines. Indeed, the study (two groups) within this review that met the WHO physical activity guidelines in their entirety implemented this approach starting from a low dose of exercise and progressing to the guidelines (Keefe et al. 2004). The authors of this study concluded that the programme was effective, mentioning that participants gained substantial general physical fitness and improved self‐efficacy in addition to lower‐limb strength and pain coping. Pragmatically, understanding an individual's baseline activity and implementing positive changes towards the WHO physical activity guidelines overtime, understanding this time may be largely dependent on the individual, appears to be a way forward for exercise‐based practice in KOA. Indeed, a large effect on health outcomes is observed even at lower levels of physical activity (i.e., improving from a sedentary baseline but not yet meeting the guidelines; Hupin et al. 2015). This means that progressive dosing of physical activity allows for initial, lower‐dose effects while aiming to move towards the established physical activity guidelines, which are shown to be effective at reducing health‐related risk when adhered to (Martinez‐Gomez et al. 2024). Thus, in a population with identified elevated risk of co‐morbidities such as cardiovascular disease (Park et al. 2023), incorporating broader health‐related considerations around exercise/physical activity appears to be a pragmatic and necessary paradigm shift.

### 4.1 Limitations

Our analysis was limited due to the poor reporting of dose and intensity within the included studies. Specifically, the small number of studies satisfying the WHO physical activity guidelines can be partially attributed to a lack of reporting of intensity within interventions. Sixty interventions including aerobic components (50.8%) and 165 (75.7%) interventions including muscle strengthening components did not report intensity. This is consistent with previous research highlighting unsatisfactory levels of reporting amongst high‐quality trials for KOA exercise interventions (Hansford et al. 2022). Moreover, this analysis was completed on an umbrella review (Varongot‐Reille et al. 2024) which identified trials until March 2022, meaning there are a number of trials to‐date not included within the analysis of this study. However, the umbrella review provides a comprehensive and representative proportion of exercise‐based RCTs in KOA and it is unlikely that the addition of a relatively small number of new trials (compared to the *n* = 266 reported in this study) will substantially influence the results.

## 5 Conclusion

The proportions of exercise interventions for KOA meeting the WHO physical activity guidelines are low. This raises concern that current evidence‐based exercise interventions for KOA may not adequately address the elevated risk factors and non‐diseases in this population. While the addition of any level of physical activity from a sedentary baseline shows health‐related improvement, using the WHO physical activity guidelines as a benchmark to work towards for people living with KOA appears a pragmatic solution in an attempt to manage health‐related risk in exercise practice. Overarchingly, a paradigm shift towards incorporating health‐based physical activity considerations in conditions such as KOA, where there appears to be no superior mode of exercise on outcomes (e.g., pain and disability) and an elevated health risk, is necessary.

## Author Contributions

**Titus E. Zhao:** data extraction (lead), formal analysis (lead), writing (equal). **Matthew D. Jones:** conceptualisation (equal), methodology (equal), investigation (equal), review and editing (equal). **Mitchell T. Gibbs:** conceptualisation (equal), methodology (equal), writing (lead), review and editing (equal).

## Conflicts of Interest

The authors declare no conflicts of interest.

## Data Availability

The data that support the findings of this study are available from the corresponding author upon reasonable request.
